# Time-Updated Phenotypic Guidance of Corticosteroids and Antibiotics in COPD: Rationale, Perspective and a Proposed Method

**DOI:** 10.3390/biomedicines11051395

**Published:** 2023-05-08

**Authors:** Alexander Jordan, Pradeesh Sivapalan, Valdemar Rømer, Jens-Ulrik Jensen

**Affiliations:** 1 Section of Respiratory Medicine, Herlev-Gentofte University Hospital, 2900 Hellerup, Denmark; 2 Department of Clinical Medicine, University of Copenhagen, 2200 Copenhagen, Denmark

**Keywords:** COPD, corticosteroids, eosinophils, biomarkers, procalcitonin, respiratory tract infections, acute exacerbation of COPD, inhaled corticosteroids

## Abstract

Chronic obstructive pulmonary disease (COPD) is a heterogeneous disease with distinct phenotypes, each having distinct treatment needs. Eosinophilic airway inflammation is present in a subset of COPD patients in whom it can act as a driver of exacerbations. Blood eosinophil counts are a reliable way to identify patients with an eosinophilic phenotype, and these measurements have proven to be successful in guiding the use of corticosteroids in moderate and severe COPD exacerbations. Antibiotic use in COPD patients induces a risk of Clostridium difficile infection, diarrhea, and antibiotic resistance. Procalcitonin could possibly guide antibiotic treatment in patients admitted with AECOPD. Current studies in COPD patients were successful in reducing exposure to antibiotics with no changes in mortality or length of stay. Daily monitoring of blood eosinophils is a safe and effective way to reduce oral corticosteroid exposure and side effects for acute exacerbations. No evidence on time-updated treatment guidance for stable COPD exists yet, but a current trial is testing an eosinophil-guided approach on inhaled corticosteroid use. Procalcitonin-guided antibiotic treatment in AECOPD shows promising results in safely and substantially reducing antibiotic exposure both in time-independent and time-updated algorithms.

## 1. Introduction

Chronic obstructive pulmonary disease (COPD) is a common disease worldwide with a substantial impact on quality of life and mortality, making it an important contributor to the global burden of disease [1,2]. It is characterized by chronic airway inflammation leading to respiratory symptoms and airway obstruction [1].

COPD is a heterogeneous disease in which the presence of emphysema, airway obstruction, excess mucus production, vascular dysfunction, and inflammation varies considerably among patients and none of these factors are particularly good predictors of symptom burden or disease development [3,4,5]. The disease occurs at the intersection of airborne insults to the lung tissue such as cigarette smoke, pollutants, allergens, genetic predisposition, pathogens, and altered immune response [4,6,7]. Therefore, the understanding of COPD has shifted from viewing the disease as a single entity to viewing it as a combination of distinct phenotypes that may differ in natural history and treatment needs. This understanding of COPD opens the door to individualized treatment. However, there is still a need for the better identification of treatable phenotypes.

Furthermore, patient phenotypes are not necessarily constant [8,9]. Even within the same phenotype, disease activity will vary based on factors such as airborne exposure to smoke, allergens, and pathogens, the control of co-morbidity, and the treatment of the lung disease [10,11,12]. Thus, it is perhaps still reductive to consider the patient as belonging to a single phenotype, and the concept should be adapted to consider the time-dependent variation in the disease. This approach could lead to the earlier identification of patients that are insufficiently treated and could act as a potential method to reduce unnecessary treatment, thus reducing drug side effects. Time-updated phenotypic guidance of therapy takes this idea and applies it in practice by continually monitoring the phenotypic state of the patient and adapting treatment as needed. Biomarkers predicting treatment response have been identified in COPD with blood eosinophils as a proven tool for corticosteroid treatment, per the growing body of high-quality evidence, acting as a model for phenotypic guided therapy [13,14]. Furthermore, procalcitonin (PCT) has shown promising potential to guide antibiotic therapy in respiratory tract infections [15], though evidence in COPD patients is still limited [16]. Other biomarkers are in the process of being used for the diagnosis and treatment of COPD exacerbation [17].

The aim of this paper is to review current evidence for time-updated phenotypic guidance of corticosteroid therapy and antibiotic-guided therapy in COPD.

## 2. Blood Eosinophils as a Treatment Response Marker

The inflammatory response in COPD involves both the innate and adaptive immune system and is mediated by neutrophils, macrophages, and CD8+ T-cells (Tc1) as well as CD4+ T-cells (Th1 and Th17) [18,19]. The presence of pro-inflammatory factors such as cigarette smoke, bacteria, or viruses in the airways stimulates the epithelium to release cytokines, causing the recruitment and activation of immune cells. This neutrophil-dominated type of inflammation responds poorly to corticosteroids [20].

Eosinophilic inflammation is characterized by elevated eosinophil counts in the blood and sputum and the activation of Th2 T-cells. This inflammatory phenotype is associated with atopic diseases such as allergies and asthma [21] and tends to respond well to treatment with corticosteroids [22,23]. However, some COPD patients exhibit increased eosinophilic inflammation, which can be a major driver of COPD. The prevalence of eosinophilic COPD varies depending on whether or not eosinophilia is measured in sputum or blood as well as does the definition used for when eosinophils are elevated. Sputum eosinophil counts of >3% are present in 28–32% [24,25] of COPD patients while blood eosinophil counts of >300 cells/μL are present in 14–24% [26,27]. Patients with a degree of eosinophilic COPD have been shown to have more frequent exacerbations [28,29,30,31], a higher risk of readmission [32] and better responses to treatment with inhaled corticosteroids (ICS) [28,33,34,35]. Sputum eosinophils have a closer association with clinical outcomes such as FEV1 or the exacerbation rate compared to blood eosinophils, though blood eosinophils are well-correlated with sputum eosinophils [36,37]. However, there are a range of challenges that come with using sputum eosinophils in clinical practice. Some patients may not be able to spontaneously produce sputum and will need induction with hypertonic saline. This process may cause bronchoconstriction in some patients and is thus unsuited to patients with severe airway obstructions such as during acute exacerbations [38]. Further, sputum induction requires a high degree of clinical training of health personnel, which makes it less feasible in many settings. Since COPD patients with eosinophilic inflammation exhibit a combination of more severe disease and better responses to treatment, this supports the earlier addition of inhaled corticosteroids to the maintenance therapy of stable COPD. This is reflected in the GOLD guidelines that favor the use of ICS when blood eosinophil counts are greater than 300 cells/μL and that favor the withdrawal of ICS when blood eosinophil counts are below 100 cells/μL [39]. However, an important consideration to be made when using eosinophils as a biomarker is that they vary over time. In stable COPD, 30–40% of patients were shown to have eosinophil counts that varied across the 300 cells/μL decision boundary over time [25,27,40]. Thus, serial eosinophil measurements may give a better picture of the inflammatory state for this subset of patients, and this opens the door to the time-updated phenotypic guidance of corticosteroid therapy and thus individualized reduction in exposure to the suspected unnecessary use of ICS.

## 3. Eosinophil-Guided OCS for Acute Exacerbations

In the treatment of acute exacerbations of COPD (AECOPD), oral corticosteroid (OCS) plays an important role. In a large meta-analysis of 16 studies (*n* = 1787), OCS has been shown to reduce the rate of treatment failure and the rate of relapse and to improve lung function and breathlessness [41]. However, no effect was seen on 30-day mortality, and OCS has numerous and severe side effects including an increased risk of infections, muscle weakness, osteoporosis, metabolic dysregulation, diabetes, cataracts, glaucoma, and gastrointestinal bleeding [42]. Therefore, some randomized trials have been conducted, examining whether or not blood eosinophils can be used to reduce OCS usage and prevent side effects (Table 1). In one trial, COPD patients with moderate or severe acute exacerbations were randomized to either receive a course of 30 mg prednisolone once daily for 14 days or to receive an eosinophil-directed therapy in which the prednisolone course was only given to patients with blood eosinophil counts of ≥2% at the time of exacerbation [43]. The eosinophil-guided regimen was found to be non-inferior to the usual standard of care and reduced corticosteroid use by approximately 50%. The STARR2 trial, which is as of now not published, but presented at the European Respiratory Society congress in 2022, examined whether or not point-of-care eosinophil measurements can be used to determine whether or not prednisolone is needed for exacerbations in general practice. In this study, patients in the intervention group received a placebo instead of prednisolone for their exacerbations if their blood eosinophil counts were below 2%, while the control group received prednisolone for 14 days irrespective of their eosinophil count. The study found no difference in antibiotic or steroid needs after 30 days [44]. The eo-Drive trial (NCT04234360) is currently ongoing with a design that randomizes patients to receive either 40 mg prednisolone once daily for 5 days or a placebo. Patients will subsequently be grouped by blood eosinophil counts with a 2% cutoff in the analysis [45]. Another ongoing trial (NCT05059873) will randomize patients with blood eosinophil counts of >2% or >300 cells/μL to a placebo or 40 mg/day oral prednisolone for 5 days in addition to standard treatment.

The aforementioned studies all look at eosinophils at the beginning of the exacerbation. This approach is limited by the fact that corticosteroid exposure may cause large changes to airway inflammation after beginning treatment, and as such, it may be individual, which patients need, e.g., in the case of 1, 2, or more days of OCS during an exacerbation. The CORTICO-COP trial accounted for this by using a true time-updated design in which the blood eosinophilia of study participants in the intervention arm was repeatedly monitored and treatment was adjusted daily, depending on whether or not blood eosinophil counts were ≥300 cells/μL [46]. No difference was observed in the primary outcome, days alive and out of hospital within 14 days, or the secondary outcome of 30-day mortality. However, the investigators found a reduced median time with corticosteroid exposure from 5 days to 2 days and a corresponding substantial reduction in the accumulated use of corticosteroids. This resulted in reduced steroid side effects such as a lower risk of worsening pre-existing diabetes and a trend towards fewer infections after 90 days.

## 4. Eosinophil-Guided ICS for Stable COPD

COPD in its stable form is treated with either long-acting beta-adrenergic agonists (LABA), long-acting muscarine antagonists (LAMA), or both [39]. For some patients, this regime is insufficient, and they still have frequent exacerbations, so the addition of ICS is necessary. While ICS is generally safe, ICS can increase exacerbations if patients are not properly selected [47]. A significant side effect of ICS is an increase in the risk of acute pneumonia and other respiratory infections such as infection with Pseudomonas aeruginosa and Hemophilus influenzae [48,49,50,51]. COPD patients with Pseudomonas infection have a highly increased risk of death [49]. Other side effects known from OCS have been proposed to occur through the systemic uptake of ICS such as in the case of diabetes [52], cataracts [53], and psychiatric effects [54,55], though the evidence is less conclusive.

While post hoc data from several large trials support the notion that ICS should be preferentially given to patients with high blood eosinophil counts [28,34,56], there is no prospective trial evidence. A 2007 trial of 82 patients used a protocol in which patients were randomized to either receive treatment according to the guidelines of the British Thoracic Society (BTS) at the time [57] or an algorithm in which bronchodilator treatment was determined by symptom severity, while corticosteroid therapy was determined by sputum eosinophil counts. Treatment was adjusted monthly for the first 6 months and then every two months for the following 6 months. This protocol led to a reduction in the frequency of severe exacerbations from 0.5 per year to 0.2 per year in the eosinophil-guided group compared to the control group. However, there was no reduction in oral corticosteroid use and no significant difference in the mean change in the daily ICS dose from the baseline [58]. As the guidelines followed in the control group did not consider eosinophils when assigning ICS treatment, it is not possible to compare the effects of time-updated therapy compared to the effect of simply assigning ICS to patients with an eosinophilic phenotype.

## 5. Future Perspectives of Eosinophil-Guided Treatment

While eosinophil-guided treatment has been shown to be a safe way to reduce corticosteroid exposure, very few of the current studies consider the significant variability of blood eosinophils over time [27]. Most have relied on a single measurement to determine the inflammatory endotype and thus treatment. While this approach is effective, it is likely that time-updated treatment guidance can further improve patient selection for corticosteroids. The currently ongoing COPERNICOS trial (NCT04481555) is the first trial of blood eosinophils as a treatment response marker for ICS with a time-updated design. It uses a protocol in which patients in the control group will receive usual treatment with ICS/LAMA/LABA while patients in the intervention group will have their ICS switched on or off every three months based on a blood eosinophil count cutoff value of 300 cells/μL with the aim of reducing corticosteroid exposure and side-effects while being non-inferior to current treatment guidelines [59].

## 6. Procalcitonin as a Biomarker in Respiratory Infections

**Figure 1:**
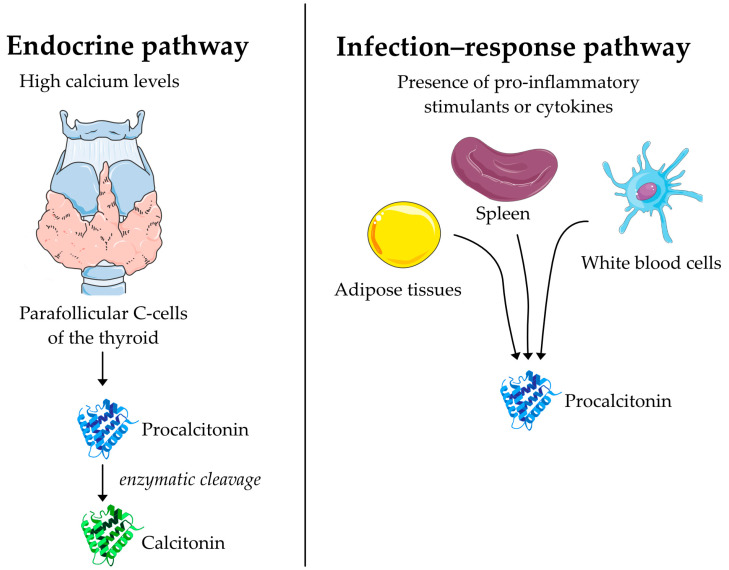
The suggested mechanism for the synthesis and secretion of procalcitonin during infection and in its endocrine function as a precursor to calcitonin. In its endocrine function, procalcitonin is created by C-cells of the thyroid as a response to high calcium levels and immediately processed into calcitonin. During infection, procalcitonin is created in a range of tissues and directly secreted into the bloodstream.

Procalcitonin (PCT) is a 116 amino acid peptide that was first discovered as the precursor to the peptide hormone calcitonin [60,61]. In its endocrine function, PCT is synthesized in the parafollicular C cells of the thyroid as a response to high calcium levels where it undergoes enzymatic processing and is secreted in the form of calcitonin acting on calcium homeostasis (Figure 1) [62]. Practically all PCT synthesized in the thyroid is converted to calcitonin, consequently leading to low levels of circulating PCT in the order of 0.01–0.05 ng/mL in the absence of disease [63]. However, during bacterial infections, PCT was found to be elevated in blood with no effect on calcitonin and declined rapidly once the infection was cleared with antibiotic therapy [64]. The mechanism of this increase in blood PCT is not entirely clear but sepsis was found to induce transcription of PCT mRNA in a range of tissues including the brain, colon, pancreas, white blood cells, spleen, and adipose tissue [65]. Patients who have undergone thyroidectomy still show an increase in PCT [66]. This led to a hypothesis that infection-related PCT does not follow the thyroid–calcitonin pathway but is instead synthesized through a distinct pathway [63]. A study on humans found that injection of endotoxin causes an increase in PCT levels [67] and experiments in cultured human mononuclear white blood cells found that interleukin 1 beta (IL1-β), IL6, tumor necrosis factor-α, and IL2, all caused an expression of PCT [68].

C-reactive protein (CRP) is a widely used biomarker for the detection and evaluation of treatment response during infections [69]. However, this biomarker has many drawbacks. It is not specific to bacterial infection and is instead more of a general inflammatory marker which can be elevated due to viral infection as well as a range of non-infectious causes such as autoimmune disease, trauma, malignancy, and necrosis [70]. CRP responds relatively quickly with a peak at about 24–72 h after stimulation but is poorly suited to evaluating when to terminate antibiotic treatment as there is a significant delay of several days from the end of the acute phase until CRP normalizes [71]. PCT has several advantages to CRP in this regard. A meta-analysis found a specificity of PCT for bacterial infection of 81% (95% CI, 67–90%) compared to that of 67% (95% CI, 56–77%) for CRP and found a sensitivity of PCT of 88% (95% CI 80–93%) compared to that of 75% (95% CI, 62–84%) for CRP. Furthermore, PCT was also more sensitive at discerning viral infections from bacterial infections, though specificities were comparable [72]. A PCT increase is detectable after only 3–4 h with a peak after 24 h and due to its short half-life of 24 h, it rapidly decreases when the infection is controlled [73]. In the context of COPD, it is also relevant that PCT is unaffected by corticosteroid treatment, while there is some evidence that CRP levels are suppressed [74,75]. This makes PCT a promising candidate for time-updated phenotypic guidance. PCT was initially tried in the context of sepsis and critical illness, but the evidence is also promising in the context of lower respiratory tract infections and COPD [76]. In COPD especially, there is a strong demand for a good biomarker to guide antibiotic treatment, as it can be difficult to discern causes of exacerbation. Better identification of bacterial exacerbations would both benefit patients by ensuring proper antibiotic treatment when needed, but also reduce antibiotic exposure, side effects, and resistance.

## 7. Procalcitonin Guidance to Reduce Antibiotic Exposure in Acute Exacerbations of COPD

Antibiotics are generously used for patients hospitalized with AECOPD, and often, antibiotic exposure exceeds guidelines [77]. Antibiotics should be used with caution, as it is associated with an increased risk of infections such as Clostridium difficile [78] infection and adds to the global threat of microbial resistance [79]. For this reason, there has been great interest in using PCT as a biomarker to reduce antibiotic exposure when treating respiratory infections (Table 2).

The randomized controlled trial proHOSP included 1359 patients with acute lower respiratory tract infections, of which 533 (39.2%) had COPD. They measured PCT on days 1, 3, and 7 of the admission and adjusted antibiotics based on whether PCT was above or below 0.25 μg/L. There were no differences in mortality, rehospitalization, or intensive care admissions, neither in the total population, nor in the subset of COPD patients. However, antibiotic prescriptions for COPD exacerbations were reduced from 69.9% to 48.7% and the mean duration of intravenous therapy was reduced from 1.9 to 1.3 days. The trial had high adherence to the protocol, which was overruled in only 10.4% of COPD exacerbations [80]. The proACT trial (*n* = 1656) used a similar design with the same thresholds for PCT, also measuring on days 1, 3, and 7 [81]. It also found no difference in adverse outcomes, but unlike proHOSP, there was no reduction in antibiotic exposure. A possible explanation of these differences could be that the protocol’s adherence was much lower in proACT compared to proHOSP. The adherence was close to 100% when the protocol recommended antibiotic treatment, but when the algorithm recommended against antibiotic treatment the adherence was generally around 60%. That was the case even with PCT <0.1, and the algorithm thus strongly recommends against antibiotics. The overall adherence for the treatment of COPD was 49.2%. The adherence was particularly low on days 3, 5, and 7. Furthermore, only 45.8% of patients in the PCT-guided group were admitted to the hospital. Only admitted patients received serial PCT monitoring, which also reduced the subset of patients for which the algorithm was used to terminate antibiotic treatment. In the proHOSP trial, days 3, 5, and 7 accounted for a large part of the difference in antibiotic exposure between the PCT-guided group and controls. Due to the low number of patients in proACT receiving serial measurements and the low protocol adherence among those, only a small subset of patients could benefit from PCT-guided antibiotic termination and the trial may thus have been insufficiently powered to show the changes among this small subset, despite the large number of patients included overall.

Several other trials have explored this type of design in COPD patients [82,83,84,85]. Most have used a threshold of 0.25 μg/L, such as the proHOSP trial, repeatedly measuring PCT and encouraging antibiotics above the threshold and discouraging it below. A few trials additionally strongly favored antibiotics for PCT >0.5 and strongly discouraged it for PCT <0.1 [80,82,83]. Some trials included only COPD patients, while others included a broader population of patients with respiratory tract infections but presented analyses in the subset of COPD patients. All trials were either open-label or single-blinded. Generally, this approach was successful in reducing antibiotic exposure with no effect on hospitalization or mortality. In one PCT-guided RCT of 120 patients hospitalized with AECOPD, there was a median reduction in antibiotic exposure of 5 days (3.5 days vs. 8.5 days) using a point-of-care PCT test and a treatment algorithm with four cut-offs [83]. However, adherence to the protocol varied across trials. One trial followed the protocol recommendations in only 61% of patients in the PCT-guided group [83]. The situation of physicians prescribing antibiotics against the algorithm is especially important, as these patients could have experienced adverse outcomes had the recommendation been followed. A 2017 meta-analysis of eight studies in COPD patients (*n* = 1062), including the aforementioned, found that PCT guidance reduced antibiotic exposure by 3.8 days (95% CI 4.32–3.35) with no differences in mortality, re-exacerbation, or readmission [16]. However, the overall quality of the evidence was low to moderate. A French trial published after this meta-analysis included 302 patients admitted to the ICU for AECOPD and found that PCT-guided therapy increased mortality from 12% in the control group to the increase of 31% in the PCT-guided group, with *p* = 0.015, indicating that PCT guidance is less appropriate for severely ill patients [86].

Two randomized trials in COPD patients used a design in which PCT was assessed only at the beginning of the exacerbation. One trial (*n* = 457) found no effect of antibiotics versus no antibiotics for patients with AECOPD and PCT < 0.1 μg/L, though the study excluded febrile patients and patients with pneumonia [87]. Another study in (*n* = 210) patients with suspected respiratory infections, 89 of which had COPD, used a threshold of 0.25 μg/L to decide whether or not to use antibiotics [88]. They found no differences in clinical outcomes and a reduction in antibiotic exposure of 40% among the COPD patients. However, sequential PCT measurements should be preferred as measuring PCT at admission does not aid decision-making on when to terminate antibiotic treatment, which is particularly relevant in the setting of infections as patient responses to treatment can vary greatly, necessitating different treatment durations.

In addition to the evidence in COPD-patients, there is strong evidence of the benefits of PCT-guided therapy when treating respiratory infections in a more general population of whom only some have COPD. A patient-level meta-analysis of 26 trials with a total of 6708 patients included examined PCT-guided antibiotics for respiratory infections in a general population of whom only some had COPD. This analysis found a significant reduction in mortality at 30 days (9% died with PCT-guided therapy vs. 10% of the controls who died; adjusted odds ratio: 0.83; 95% CI: 0.70 to 0.99) and a reduction in antibiotic exposure from 8.1 days to 5.7 days (95% CI: −2.71 to −2.15) with PCT-guided therapy [89].

A limitation of PCT is that it, like CRP, can respond to non-infectious causes such as trauma, malignancy, or kidney disease. Consequently, some patients have higher baseline levels and respond differently to infections, making it difficult to create universal cut-off levels [90]. This consideration is also present in COPD patients which may have chronic inflammation and sometimes bacterial colonization, potentially influencing baseline PCT levels [91]. Furthermore, a sub-study of a clinical trial examining the addition of doxycycline to prednisolone in the treatment of AECOPD found that patients with PCT <0.1 ug/L still experienced beneficial effects with antibiotics [92]. Thus, there is still some uncertainty as to how well the results from the selected populations and controlled circumstances of clinical trials will translate to day-to-day practice.

**Table 2:** Overview of trials examining PCT-guided therapy in COPD patients.

First Author, Year	*n* with COPD	Population	Intervention Design	Results among COPD Patients with PCT-Guided Therapy
Christ-Crain, 2004 [82]	60/243	Emergency department patients with suspected lower respiratory tract infections.	Open-label. PCT measured at admission and after 6–24 h. PCT cut-offs at 0.1, 0.25, and 0.5 μg/L. *	A 56% reduction in antibiotics prescription. No difference in risk of death, readmission, or future exacerbations.
Corti, 2016 [83]	120	Patients admitted with AECOPD.	Open-label. PCT measured sequentially at admission and on days 3, 5, and 7. PCT cut-offs at 0.1, 0.25, and 0.5 μg/L. *	Antibiotic exposure reduced from 8.5 days (IQR 1–11) to 3.5 days (IQR 1–10). No difference in composite endpoint of death, rehospitalization and ICU admission within 28 days.
Daubin, 2018 [86]	302	COPD patients admitted to ICU with AECOPD	Open-label. PCT measured sequentially at admission and on days 3 and 5. PCT cut-offs at 0.1, 0.25, and 0.5 μg/L. *	PCT-guided therapy increased mortality from 12% in the control group to 31% in the PCT-guided group.
Huang, 2018 [81]	524/1656	Emergency department patients with suspected lower respiratory tract infections.	Open-label. PCT measured sequentially at admission and on days 3, 5, 7. PCT cut-offs at 0.1, 0.25, and 0.5 μg/L. *	No difference in antibiotic prescriptions. No differences in composite outcome of death, ICU admission and readmission.
Kristoffersen, 2009 [88]	89/120	Patients admitted with suspected lower respiratory tract infections.	Open-label. PCT measured at admission. PCT cut-off at 0.25 μg/L. **	Antibiotic exposure reduced from 6.8 (95% CI 5.9–7.7) days to 5.1 (4.4–6.0) days among all patients included. No difference in ICU admission or death.
Schuetz, 2009 [80]	533/1359	Emergency department patients with suspected lower respiratory tract infections.	Open-label. PCT measured at admission, discharge and on days 3, 5 and 7. PCT cut-offs at 0.1, 0.25, and 0.5 μg/L. *	Antibiotic prescription rates reduced from 69.9% to 48.7%. No difference in composite outcome of death, ICU admission, reinfection, abscess formation, and empyema within 30 days.
Stoltz, 2007 [84]	226	Patients admitted with AECOPD.	Open label. PCT measured at admission. Antibiotics discouraged at PCT of <0.1 μg/L and encouraged at PCT of >0.25 μg/L.	Antibiotic prescription rate reduced from 72% to 40%. No difference in composite of self-reported symptoms and death.
Verduri, 2015 [85]	183	Patients admitted with AECOPD.	Open-label. PCT measured on days 1,2 and 3. Antibiotics stopped on day 3 if all measurements were <0.1 μg/L, or if all measurements were <0.25 and the patient was clinically stable.	In total, 45 of 88 patients in the PCT-guided group received treatment for 3 days rather than 10. No difference in re-exacerbation rate.
Wang, 2016 [87]	194	Patients admitted with AECOPD and PCT of <0.1 μg/L.	Open-label. PCT of <0.1 was a criterium for inclusion. Randomized to either antibiotics or no antibiotics.	No differences in self-reported symptoms, length of stay, ICU admission, mortality or rehospitalization.

* Antibiotics strongly discouraged at PCT < 0.1, discouraged at PCT < 0.25, encouraged at PCT > 0.25, and strongly encouraged at PCT > 0.5. ** Antibiotics discouraged at PCT < 0.25, and encouraged at PCT > 0.25.

## 8. Conclusions

There is an increasing body of evidence that supports that time-updated phenotypical treatment guidance can carry some pronounced benefits for patients and societies. Procalcitonin is a promising candidate for guiding the antibiotic therapy of AECOPD with evidence from several large well-designed trials using a time-updated design. Blood eosinophil measurements have proven to be successful in guiding the use of oral corticosteroids in moderate and severe COPD exacerbations, and post hoc analyses from large trials indicate that it could be a viable biomarker to use to guide ICS treatment as well. However, unlike procalcitonin, few studies account for the temporal variability of eosinophils levels. The CORTICO-COP trial has shown that daily monitoring of blood eosinophils is a safe and effective way to reduce OCS exposure and side effects for acute exacerbations. No time-updated studies have been completed for treatment with ICS in the stable phase of COPD, but an ongoing trial is testing this approach.

## Figures and Tables

**Table 1:** Overview of trials examining eosinophil-guided therapy in COPD patients with acute exacerbations.

First Author, Year	*n*	Population	Design	Results
Bafadhel, 2012 [43]	164	Patients admitted with AECOPD.	Double-blinded. Usual care compared with corticosteroids given only to patients having blood eosinophil counts of >2% at admission.	Only 49% of exacerbations in the eosinophil-guided group received corticosteroids. Similar rates of treatment failure in the two groups.
Ramakrishnan, 2022 (conference paper) [44]	203	Patients with AECOPD treated in general practice.	Double-blinded. Usual care compared with corticosteroids given only to patients having blood eosinophil counts of >2% at the start of treatment.	A total of 34 of the 102 patients in the eosinophil-guided group received a placebo. No differences in rates of treatment failure.
Sivapalan, 2019 [46]	318	Patients admitted with AECOPD	Open-label. Usual care compared with eosinophil guided using daily measurements and a 300 cells/μL cutoff for initiating or terminating treatment	Mean antibiotic exposure duration was reduced from 5 to 2 days with eosinophil-guided therapy. No difference in days alive and out of hospital within 14 days or in 30-day mortality.
